# Effects of Dietary Crude Protein Level on Growth Performance, Carcass Traits, Meat Quality, and Fatty Acid Composition of Ningxiang Finishing Pigs

**DOI:** 10.3390/ani15202950

**Published:** 2025-10-11

**Authors:** Xianglin Zeng, Yan Tang, Wenzhi Liu, Zhaobin Wang, Pengfei Huang, Qiye Wang, Huansheng Yang

**Affiliations:** Hunan International Joint Laboratory of Animal Intestinal Ecology and Health, Laboratory of Animal Nutrition and Human Health, College of Life Sciences, Hunan Normal University, Changsha 410081, China; zxlin@hunnu.edu.cn (X.Z.); 19015850349@163.com (Y.T.); 202370142924@hunnu.edu.cn (W.L.); wzb@hunnu.edu.cn (Z.W.); pengfeihuang@hunnu.edu.cn (P.H.)

**Keywords:** Ningxiang finishing pigs, dietary crude protein, carcass fat percentage, meat quality

## Abstract

**Simple Summary:**

This study examined the effects of varying crude protein (CP) levels on growth performance, carcass traits, meat quality, and fatty acid composition in Ningxiang finishing pigs. The results revealed that low-protein (LP) diets reduced lean meat percentage and increased fat deposition, while simultaneously improving meat quality and fatty acid profiles. Furthermore, LP diets facilitated fat accumulation by suppressing hepatic tryptophan–niacin metabolism and inhibiting the AMPK signaling pathway in muscle, thereby influencing carcass traits and meat quality. These findings offer valuable insights into optimizing dietary protein strategies to enhance growth performance and meat quality in indigenous pig breeds.

**Abstract:**

This study investigated the effects of different crude protein (CP) levels on growth performance, carcass traits, meat quality, and fatty acid composition in Ningxiang finishing pigs. A total of 200 pigs (52.52 ± 0.41 kg) were assigned to five dietary treatments: high-protein (HP, 15.56%), moderate-high-protein (MHP, 13.99%), moderate-protein (MP, 12.94%), moderate-low-protein (MLP, 11.90%), and low-protein (LP, 10.31%). Feeding the MLP diet significantly improved average daily gain (ADG) compared to HP and LP diets (*p* < 0.05). Pigs fed the MP diet had higher lean meat percentage than those on the LP diet (*p* < 0.05), while both HP and MP diets reduced carcass fat percentage (*p* < 0.05). The LP diet significantly increased a*, pH_45min_, intramuscular fat (IMF), and C18:1n9c, while decreasing C23:0 and C20:3n6 in the longissimus dorsi muscle (*p* < 0.05). Liver metabolomics revealed that the LP diet inhibited nicotinate and nicotinamide metabolism (*p* < 0.05), accompanied by downregulation of genes related to the tryptophan–niacin metabolism and upregulation of genes involved with hepatic lipogenesis (*p* < 0.05). In muscle, the LP diet inhibited AMPK signaling via decreased p-AMPK expression, leading to increased IMF content (*p* < 0.05). In summary, the optimal growth performance of Ningxiang finishing pigs was achieved with a CP level of 11.90%. Additionally, the LP diet enhanced meat quality by modulating hepatic niacin metabolism and AMPK signaling.

## 1. Introduction

The Ningxiang pig is a local pig breed in China, known for its high reproduction rate, tolerance to roughage, and strong disease resistance [1]. Compared with common pig breeds on the market, Ningxiang pigs have stronger fat deposition and better meat quality [2,3]. With increasing consumer demand for high-quality pork, Ningxiang pigs are gaining prominence in the premium pork market due to their superior meat quality and favorable sensory attributes [4,5,6]. As a prototypical fat-type indigenous Chinese breed, Ningxiang pigs demonstrate distinct growth patterns, fat deposition characteristics, and differential nutritional requirements when compared with lean-type porcine varieties.

Crude protein (CP) in swine diets provides the animals with the amino acids (AAs) they need for optimal growth and development. Excessively high CP levels are not necessarily beneficial to pig growth, but will increase feed costs and cause a waste of protein resources [7]. Appropriate reduction in dietary CP levels and supplementing essential amino acids (EAAs) have been demonstrated to decrease nitrogen excretion and reduce feed costs and environmental pollution without impairing the growth performance of the pigs [7,8,9]. However, excessive reduction in dietary CP levels may negatively impact growth performance and impair animal health [7,10,11]. Several studies have aimed at reducing CP levels in the diets of crossbred (Duroc × Landrace × Yorkshire) finishing pigs [12,13,14], but few studies have investigated the optimal CP levels for fat-type finishing pigs. Therefore, investigating the effects of crude protein levels on the growth performance of Ningxiang pigs is highly significant for optimizing feeding strategies in fat-type pigs.

Intramuscular fat (IMF) content and fatty acid composition are the primary factors influencing meat quality [15]. IMF is directly related to tenderness, juiciness, marbling, and flavor development of cooked meats [16]. Reducing dietary CP levels can increase fat percentage and IMF content while also improving the fatty acid composition during the finishing period [13,17]. However, excessive fat accumulation, specifically increased carcass fat percentage, may compromise profitability. This phenomenon necessitates further investigation into how LP diets modulate carcass fat deposition and intramuscular fat content to enhance meat quality and reduce production costs.

This study aimed to evaluate the effects of different CP levels on the growth performance, carcass traits, meat quality, and fatty acid composition of Ningxiang finishing pigs. Additionally, it sought to elucidate the potential mechanisms by which CP levels regulate fat deposition, providing valuable insights for optimizing feeding strategies in fat-type pigs.

## 2. Materials and Methods

### 2.1. Animals and Experimental Design

The animal trials were conducted at the experimental pig farm of Hunan ChuWeiXiang Agriculture and Animal Husbandry Co., Ltd. (Changsha, China). A total of 200 Ningxiang pigs (52.52 ± 0.41 kg of average body weight) were randomly divided into five groups (half barrow and half female), with 5 replicate pens in each group and 8 pigs in each replicate. Five experimental diets (formula details are shown in Table 1) were formulated according to the Chinese feed composition and nutritional value table [18] and the Nutrient Requirements of Chinese Pigs [19]. The target CP levels were 15.00%, 14.00%, 13.00%, 12.00%, and 11.00%, decreasing in a stepwise manner using an arithmetic progression design. Chemical analysis of the experimental diets showed actual CP contents of high protein (HP, CP: 15.56%), medium high protein (MHP, CP: 13.99%), medium protein (MP, CP: 12.94%), medium low protein (MLP, CP: 11.90%), and low protein (LP, CP: 10.31%). The slight discrepancies between formulated and measured values were attributable to natural variation during feed preparation. The HP diet was a diet based on corn and soybean meal, which served as the control diet. The MHP, MP, MLP, and LP diets achieved amino acid balance among the groups by gradually reducing soybean meal while adding six crystalline amino acids (lysine, threonine, tryptophan, methionine, valine, and isoleucine). The amino acid standardized intestinal digestibility (SID) values of the treatment groups remained consistent, while the net energy (NE) levels remained consistent, and the amino acids and net energy met the requirements of the Nutrient Requirements of Chinese Pigs [19]. Additionally, GE was determined using the bomb calorimetric method [20], while crude protein (CP) was measured using the Kjeldahl method [21]. The experimental diets were formulated at Hunan ChuWeiXiang Agriculture and Animal Husbandry Co., Ltd. and provided to the pigs in pelleted form. The experiment lasted for 61 days, during which all pigs had unlimited access to water and designated feed. Feed consumption of Ningxiang pigs in each pen was recorded daily, and pigs were weighed by pen at the beginning and end of the experiment. At the end of the experiment, seven pigs from each of the HP, MP, and LP treatment groups (*n* = 7 per group) were randomly selected from individuals whose body weights (BWs) were closest to the respective group mean to serve as representative biological replicates. These pigs were deprived of food for 12 h and subsequently euthanized via electrical stunning. Samples of the longissimus dorsi muscle (LM) and liver were collected from the carcasses and stored at −80 °C for further analysis. LM and abdominal fat were harvested following the method described by Wang et al. [1], after which adipocyte morphology was observed and meat quality was assessed. LM samples were stored at −20 °C, while abdominal fat was fixed in 10% formaldehyde solution.

### 2.2. Slaughter Performance

Following slaughter, Ningxiang pigs underwent standardized processing involving removal of hair, head, hooves, tail, and visceral organs (retaining suet and kidneys). Carcass weight was recorded post-evisceration. The left carcass was then suspended upside down, and backfat thickness was measured using a vernier caliper at three anatomical landmarks: the thickest point of the shoulder, the last rib, and the lumbosacral region. The average backfat thickness was calculated as the mean of these three measurements. The left leg was vertically incised between the first to last and the second to last lumbar vertebrae and weighed, and the leg-to-hip ratio (%) = (hip weight of the left leg/left carcass weight) was calculated according to the method of Zeng et al. [22]. The straight length and oblique length of the carcass were determined according to the established protocol [1]. The carcass was divided into four components: skin, bone, fat, and lean meat (including intramuscular fat, cartilage, and tendons, all classified as lean meat). Each component was weighed, and its proportions relative to the whole carcass were calculated.

### 2.3. Meat Quality

The pH of LM was measured at 45 min and 24 h after death using a pH meter (PH-828, SMART SENSOR, Dongguan, China) with automatic temperature compensation at 4 °C ambient temperature and calibrated in standard buffer solutions of pH 4.0 and 7.0 before measurement. A portable colorimeter (CR-410, Konica Minolta, Chiyoda, Japan; illuminant D65; standard observer 2°; measuring diameter 50 mm) was calibrated with a standard white plate to evaluate the LM color of Ningxiang finishing pigs 45 min after slaughter, including: L* (brightness), a* (redness), and b* (yellowness).

### 2.4. Determination of Fatty Acid Composition

To determine the fatty acid composition, add approximately 0.5 g of freeze-dried LM tissue to 4 mL of benzene–petroleum ether (1:1, *v*/*v*), seal, and extract it for 24 h. Then, add 4 mL KOH (0.4 M) and 4 mL ddH_2_O to the extracted lipids for stratification, take 2 mL of the upper solution, and add 1 g of anhydrous copper sulfate to absorb the water in the sample. Add n-hexane to the sample in a ratio of 1:4, then pass it through a 0.22 μm filter membrane and put it on the machine. Use a 7890B gas chromatograph (Agilent, Santa Clara, CA, USA) to separate and determine the fatty acid methyl esters in the samples. Gas chromatography conditions: chromatographic column sp-2560 (100 m × 0.25 mm × 0.2 μm), equipped with hydrogen flame ionization detector, temperature is 280 °C; heating program initial temperature is 140 °C, hold for 5 min, raise temperature to 220 °C at 3 °C/min, and hold for 40 min; injection volume 1 μL; split ratio 20:1, carrier gas is nitrogen, flow rate 0.8 mL/min. The fatty acids were identified by comparing the retention times of the peaks with those of known standards (47885-u, Sigma-Aldrich, Saint Louis, MO, USA), and the percentage of each fatty acid relative to total fatty acids was used as a phenotype for further analyses.

### 2.5. Adipocyte Morphological Analysis

To study adipocyte morphology, fixed adipose tissue was embedded in paraffin. Three cross-sections with a thickness of 4 μm were taken from each abdominal fat section and stained with hematoxylin and eosin (H&E). Area, diameter, and perimeter were determined at 40× magnification using an orthochromatic fluorescence microscope (Version 4.12, Leica, London, UK), and density was calculated. About 100–200 adipocytes per sample were evaluated.

### 2.6. Untargeted Metabolomic Analysis of Liver

Five liver samples were selected from each treatment group and prepared according to the methodology described by Wang et al. [23]. LC-MS analysis was conducted at Shanghai Bioprofile Technology Co., Ltd., Shanghai, China. Raw data underwent peak detection and alignment using MSDIAL ver.4.18 software. To ensure data quality and integrity, ion peaks with missing values exceeding 50% within any group were removed from the dataset and excluded from subsequent statistical analyses. The positive and negative ion data were normalized based on the total peak area, respectively. Positive and negative ion peaks were integrated, and pattern recognition was performed using R ver.4.2.0 software. Following unit variance scaling (UV) and preprocessing, further data analyses were conducted.

### 2.7. RNA Extraction and Real-Time Quantitative PCR

Total RNA was extracted from the LM sample using RNAiso Plus (9109, TaKaRa, Beijing, China). RNA was transcribed into cDNA according to the instructions of the reverse transcription kit (RR047A, TaKaRa, China) and diluted at a ratio of 1:5. Primers (Table 2) were designed using Primer-BLAST and synthesized by Sangon Biotech (Shanghai, China). The expression levels of mRNA of the selected genes were evaluated using RT-qPCR instrument (ABI 7900HT, Applied Biosystems, Foster City, CA, USA). The 2^−ΔΔCt^ method was used to calculate the relative mRNA expression level of the target gene [24].

### 2.8. RNA Sequencing

Total RNA was extracted from LM samples, and its concentration and purity were evaluated using NanoDrop 2000 (Thermo Fisher Scientific, Waltham, MA, USA). Libraries were prepared using the Illumina^®^ Stranded mRNA Prep, Ligation Kit (Illumina, San Diego, CA, USA) following the manufacturer’s protocol, and subsequently sequenced on the Illumina NovaSeq 6000 platform (Illumina, USA). Sequencing reads were aligned to the reference porcine genome (Sscrofa 11.1, available for download from Ensembl, Hinxton, UK) using default parameters of Version 2.1.2. Then, weighted gene correlation network analysis (WGCNA) was performed to divide genes with similar expression patterns into a module, thereby finding gene modules with similar phenotypes.

### 2.9. Western Blot

Protein extracts were obtained from LM tissues using RIPA lysis buffer (P0013B, Beyotime, Nantong, China) combined with protease inhibitors (PMSF, Beyotime ST056, Beyotime, China) following the manufacturer’s protocol. Protein concentration was determined using a kit (ZJ102, EpiZyme, Beijing, China), and each sample was adjusted to equivalent concentrations with 1× loading buffer (BL502A, Biosharp, Hefei, China) and then mixed with 5× loading buffer and placed in a metal bath at 100 °C for 10 min to ensure protein denaturation. Following gel electrophoresis, membrane transfer, and blocking procedures, the membranes were incubated overnight at 4 °C with the following primary antibodies: phospho-AMPKα (2535S; Cell Signaling Technology, Danvers, MA, USA) and GAPDH (60004; Proteintech, Wuhan, China), both diluted at 1:3000. Subsequently, the membranes were incubated for 1 h at room temperature with an HRP-conjugated anti-rabbit IgG secondary antibody (7074P2; Cell Signaling Technology, USA), diluted 1:10,000. The blots were visualized utilizing an Omni-ECL detection kit (SQ202, EpiZyme, China).

### 2.10. Statistical Analyses

Growth performance was measured at the pen level, while individual pigs served as the experimental units for other measurements. All data were analyzed using SPSS ver. 26.0 statistical software. One-way analysis of variance (ANOVA) was applied to assess the effects of dietary protein levels, and linear and quadratic regression analyses were conducted to evaluate differences between treatments. If ANOVA detected significant differences, post hoc comparisons of treatment means were performed using Duncan’s multiple range test. For RT-qPCR and Western blotting, unpaired Student’s *t*-tests were used. Results are presented as mean ± SEM. The threshold for statistical significance was established at *p* < 0.05.

## 3. Results

### 3.1. Effects of Dietary CP Levels on Growth Performance, Carcass Traits, and Meat Quality of Ningxiang Finishing Pigs

As presented in Table 3, the MLP diet increased the ADG of Ningxiang finishing pigs compared to the HP and LP diets (*p* < 0.05). A significant quadratic relationship was observed between dietary CP level and ADG (Quadratic, *p* < 0.05), with the highest ADG occurring in pigs fed the MLP diet. Furthermore, there were no significant differences in ADFI and G:F among the treatments with varying dietary CP levels (*p* > 0.05).

The carcass characteristics of Ningxiang finishing pigs are shown in Table 4. The fat percentage of carcasses was significantly increased with the LP diet compared to the HP and MP diets (*p* < 0.05). Moreover, the fat percentage of carcasses increased (*p* < 0.05, Linear) with a lower dietary CP level. The lean meat percentage was significantly decreased in pigs fed the LP diet compared to those fed the MP diet (*p* < 0.05). A significant quadratic relationship was observed between dietary CP level and lean meat percentage (Quadratic, *p* < 0.05), with the highest lean meat percentage recorded in pigs fed the MP diet.

The quality of the LM meat of Ningxiang finishing pigs is shown in Table 5. a*, pH_45min_, and IMF content of the LP diet were increased compared with those of the HP and MP diets. (*p* < 0.05). Meanwhile, a*, pH_45min_, and IMF content increased with decreasing dietary CP levels (*p* < 0.05, Linear). A significant quadratic relationship was observed between dietary CP level and IMF content (Quadratic, *p* < 0.05), with the lowest IMF content recorded in pigs fed the MP diet.

### 3.2. Effect of Dietary CP Levels on Fatty Acid Composition of LM in Ningxiang Finishing Pigs

The fatty acid composition of the LM from Ningxiang finishing pigs is presented in Table 6. Compared with the MP group, the LP group contained a lower (*p* < 0.05) proportion of tricosanoic acid (C23:0). Compared with the MP group, the LP group had a higher (*p* < 0.05) proportion of oleic acid (C18:1n9c). Compared with the HP and MP groups, the LP group had a higher (*p* < 0.05) proportion of total monounsaturated fatty acids (ΣMUFAs). Moreover, the LP group significantly reduced (*p* < 0.05) the cis-8,11,14-Eicosatrienoic acid (C20:3n6) proportion compared with the HP and MP groups. The proportions of C20:3n6 decreased with the decrease in dietary CP level (Linear, *p* < 0.05), and ΣMUFA increased with the decrease in CP level (Linear, *p* < 0.05). A significant quadratic relationship was observed between dietary CP level and the proportions of C17:0, C23:0, C18:1n9c, and C20:3n6 (Quadratic, *p* < 0.05). The lowest proportions of C17:0 and C18:1n9c were observed in pigs fed the MP diet, while the highest proportions of C23:0 and C20:3n6 were also recorded in pigs fed the MP diet.

### 3.3. Effect of Dietary CP Levels on Adipocyte Morphology in Ningxiang Finishing Pigs

**Figure 1:**
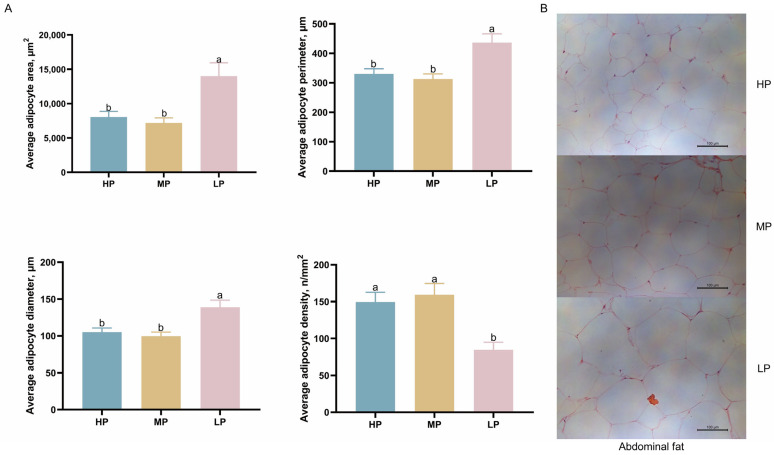
Effects of dietary CP levels on abdominal fat cell morphology of Ningxiang finishing pigs. (**A**) Area, perimeter, diameter, and density of fat cells. (**B**) Representative images of abdominal fat cell morphology. 200× magnification; scale bar, 100 μm. Error bars indicate the mean ± SEM (*n* = 5; one-way ANOVA). Means without a common letter differ significantly by Duncan’s multiple range test (*p* < 0.05).

The morphology of abdominal fat cells in Ningxiang finishing pigs is shown in Figure 1. Adipocyte area, perimeter, and diameter were significantly increased in the LP diet compared with the HP and MP diets (*p* < 0.05). Furthermore, the LP diet significantly reduced adipocyte density compared with the HP and MP diets (*p* < 0.05).

### 3.4. Effect of Dietary CP Levels on Liver Metabolomics in Ningxiang Finishing Pigs

**Figure 2:**
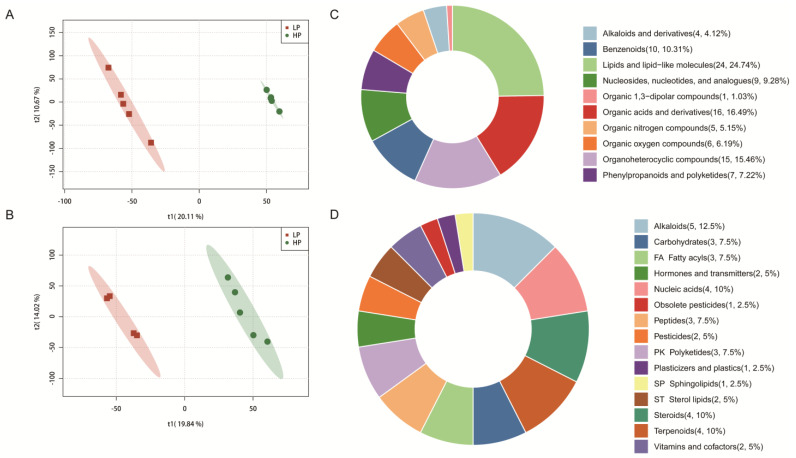
Effects of dietary CP level on the liver metabolome of Ningxiang finishing pigs. (**A**) Partial least squares discriminant analysis (PLS−DA) based on the positive ion table. (**B**) PLS−DA based on the negative ion table. Differential metabolite classification based on HMDB (**C**) and KEGG (**D**).

Figure 2A,B show the partial least squares discriminant analysis (PLS-DA) of the liver metabolic profile. There were differences in the liver metabolic characteristics between the HP and LP groups in both positive and negative ion modes. Notably, there was no overlap in the data between the two groups, indicating distinct metabolic profiles.

**Figure 3:**
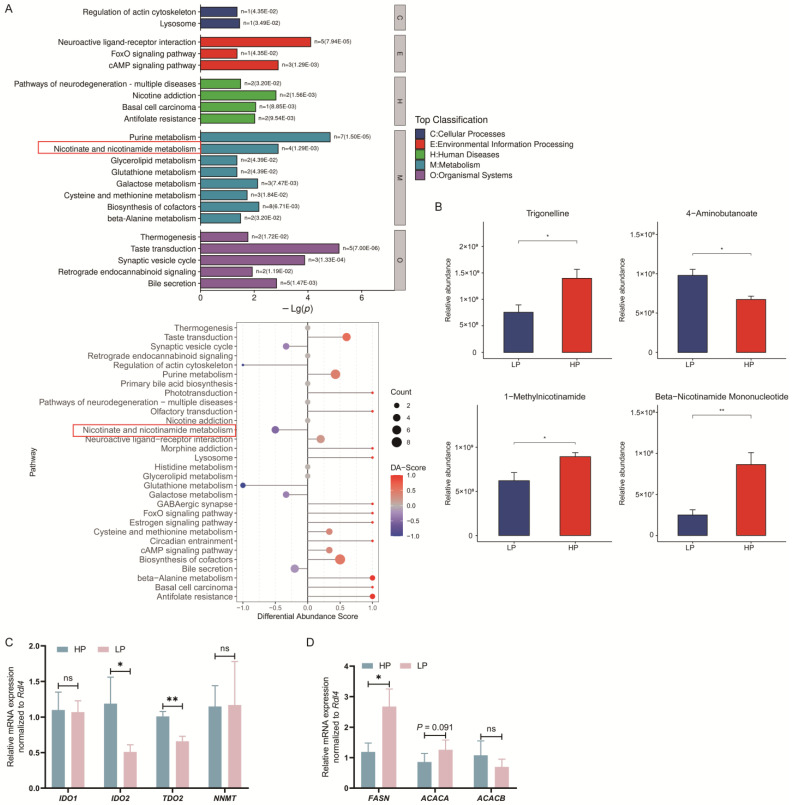
Effects of dietary crude protein levels on metabolite enrichment and gene expression related to nicotinate and lipogenesis in Ningxiang finishing pigs. (**A**) Differential metabolite KEGG enrichment analysis. (**B**) Differential metabolites in nicotinate and nicotinamide metabolism. (**C**) RT-qPCR analyzed the mRNA levels of related genes in the atryptophan–nicotinic acid pathway. (**D**) RT-qPCR analyzed the mRNA levels of lipogenic genes. Error bars indicate the mean ± SEM (*n* = 5). *, *p* < 0.05, **, *p* < 0.01, ns, *p* > 0.10 by unpaired Student’s *t*-test.

A total of 179 metabolites were identified to be different between the HP and LP groups (Figure 2C,D). Among these, 97 metabolites were sourced from the HMDB, and 40 metabolites were sourced from the KEGG. The classification of compounds based on the HMD revealed that the identified metabolites were primarily divided into 10 classes. Notably, lipids and lipid-like molecules accounted for the highest proportion, comprising 24.74% of the total metabolites (Figure 2C). Additionally, the classification based on the KEGG showed that the metabolites were categorized into 15 distinct classes (Figure 2D). Subsequent metabolic pathway analysis conducted on the differential metabolites from the livers revealed that the LP diet significantly altered 22 metabolic pathways in Ningxiang finishing pigs (Figure 3A). Among them, the taste transduction, neuroactive ligand-receptor interaction, purine metabolism, cAMP signaling pathway, synaptic vesicle cycle, and nicotinate and nicotinamide metabolism are the six most significantly enriched KEGG pathways. In addition, we also found that the metabolism of nicotinate and nicotinamide was downregulated in the LP group. Compared with the HP group, the LP group had four differential metabolites related to nicotinic acid and nicotinamide metabolism, among which trigonelline, 1-methylnicotinamide, and beta-nicotinamide mononucleotide were reduced, and 4-aminobutanoate was increased (Figure 3B).

### 3.5. Effect of Dietary CP Level on mRNA Expression of Genes Related to Tryptophan–Niacin Metabolism and Lipogenesis in Liver of Ningxiang Finishing Pigs

The mRNA expression of tryptophan–niacin-related genes, such as *IDO2* and *TDO2*, was downregulated in the LP diet compared with the HP diet (*p* < 0.05; Figure 3C). Since niacin regulates lipogenesis, we also measured the expression of lipogenic genes in the liver. *FASN* mRNA expression was upregulated in the LP diet compared with the HP diet (*p* < 0.05; Figure 3D). *ACACA* mRNA expression tended to be upregulated in the LP diet compared with the HP diet (*p* = 0.091; Figure 3D).

### 3.6. WGCNA

**Figure 4:**
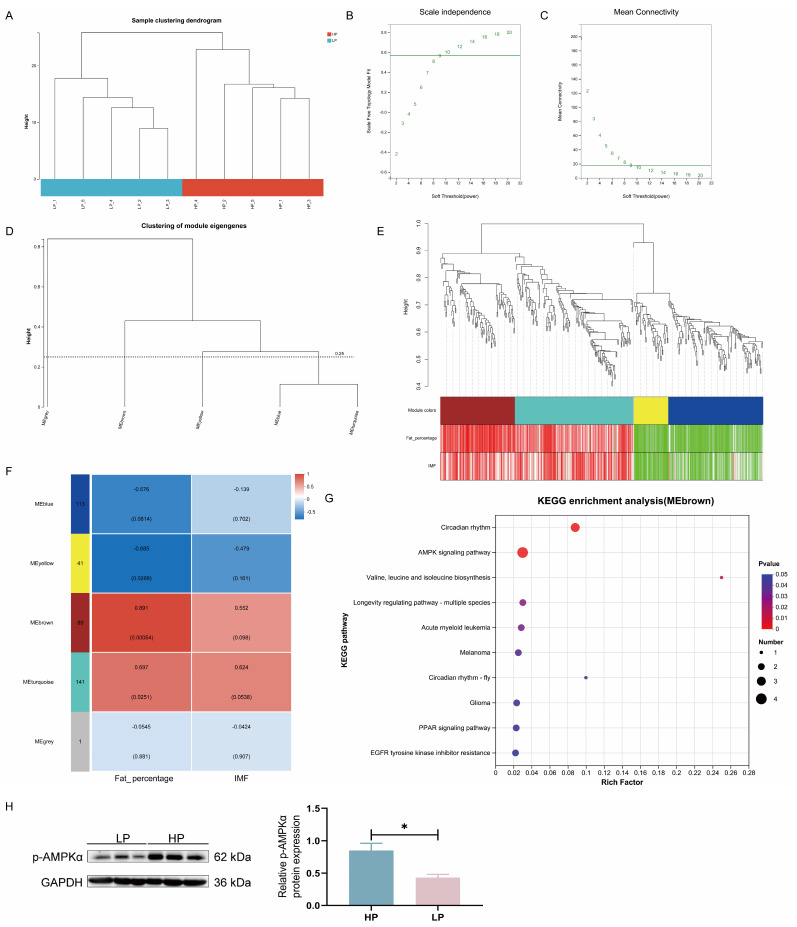
WGCNA was employed to identify and validate gene co-expression networks associated with carcass fat percentage and muscle IMF content in Ningxiang finishing pigs. (**A**) Sample cluster diagram. A network topology analysis for adjacency matrices with different soft threshold powers. Green numbers indicated the soft-threshold power corresponding to the correlation coefficient square value in (**B**) and mean connectivity in (**C**). (**D**) The gene cluster tree was constructed according to the correlation between gene expression levels. The similarity degree selected was 0.75, and the minimum number of genes selected for analysis was 1, in this analysis. (**E**) Correlation plot of genes and phenotypes within modules identified by WGCNA. (**F**) Module–trait associations. Each row corresponds to a module eigengene, and each column to a trait. Each cell contains the corresponding correlation and *p*-value. The legend represents the correlation coefficient: the colors red and blue represent positive and negative correlations, respectively. (**G**) KEGG-enriched pathway map of the brown module. (**H**) Western blotting was used to detect the p-AMPKα protein level in LM of Ningxiang finishing pigs. Error bars indicate the mean ± SEM (*n* = 3). *, *p* < 0.05 by unpaired Student’s *t*-test.

Ten Ningxiang finishing pig LM samples were clustered by WGCNA to construct a co-expression network (Figure 4A). When the lowest soft threshold power was set to 9, the scale-free topology fit index (R^2^) reached 0.57, confirming the establishment of a scale-free network (Figure 4B,C). A gene clustering tree was generated based on pairwise correlations of gene expression levels, and the dendrogram was subsequently partitioned into five discrete gene modules (Figure 4D,E). By looking for correlations between characteristic genes and trait characteristics (fat percentage and IMF content), the brown module is most strongly correlated with fat percentage and is positively correlated (Figure 4F). The brown module also shows a positive correlation trend with the IMF content. Furthermore, fat percentage was positively correlated with the turquoise module and negatively correlated with the yellow module. The fat percentage showed a negative correlation trend with the blue module, while the IMF content and the turquoise module showed a positive correlation trend.

To further understand the functions of genes in the brown module, we performed KEGG functional enrichment analysis (Figure 4G). The results show that they are mainly enriched in the following: circadian rhythm, AMPK signaling pathway, valine, leucine, and isoleucine biosynthesis, longevity regulatory pathway—multiple species, acute myeloid leukemia, melanoma, PPAR signaling pathway, circadian rhythm—fly, glioma, and EGFR tyrosine kinase inhibitor resistance. AMPK is an energy sensor that inhibits lipogenesis and promotes lipolysis. Compared with Ningxiang finishing pigs fed the HP diet, the p-AMPKα level in LM of Ningxiang finishing pigs fed the LP diet was decreased (*p* < 0.05; Figure 4H), indicating that the LP diet inhibited AMPK signaling.

## 4. Discussion

This study aims to explore the effects of different CP levels on the growth performance of Ningxiang finishing pigs under the condition of six balanced kinds of EAAs in the diet, and to explore the effects of changes in CP levels on the carcass traits and meat quality of Ningxiang finishing pigs and their potential mechanisms. Our data shows that the dietary CP level of 11.90% significantly increased the ADG of Ningxiang finishing pigs. These suggest that moderately reducing dietary CP, together with EAA supplementation, can enhance growth performance in Ningxiang pigs. However, excessive reductions in CP appeared to compromise these benefits, as indicated by the optimal growth performance observed in the MLP group. This finding is consistent with previous studies demonstrating the existence of a threshold CP level below which optimal growth cannot be maintained, even with sufficient EAA supplementation [7,25,26]. Notably, although HP diets can theoretically supply sufficient amino acids, an excess of non-limiting amino acids may disrupt the amino acid balance, thereby impairing growth performance [27].

We will now further evaluate the effects of CP levels on carcass traits and meat quality, the HP, MP, and LP groups (representing the extremes and the intermediate of dietary CP levels, respectively). This design enabled us to capture the overall trends of protein effects while reducing the number of animals required for post-slaughter measurements. Carcass traits are a direct reflection of the meat production performance of Ningxiang pigs and are directly related to the economic benefits of farmers [1,28,29]. Several studies have reported the effects of different CP levels and AA supplementation methods on the carcass traits of the pigs and showed inconsistent results [7,30,31]. In this study, compared with HP and MP diets, the fat percentage of Ningxiang finishing pigs that were fed LP diets increased significantly. Furthermore, the LP diet significantly reduced lean meat percentage compared with the MP diet. These findings are consistent with certain prior studies indicating that moderate reductions in dietary CP levels, combined with CAA supplementation, do not adversely affect carcass traits, whereas excessive CP reduction may result in increased carcass fat percentage [8,30,31,32]. In the present study, all experimental diets were isoenergetic in NE content. Consequently, carcass composition variations cannot be attributed to energy availability. The reduced lean meat percentage in the LP group is primarily attributed to an energy-to-nitrogen imbalance [7]. These findings suggest that, when HP diets are used as the reference, moderate reduction in dietary CP does not adversely affect carcass traits, excessive CP restriction compromises lean tissue accretion and promotes nutrient repartitioning toward adipose deposition.

Appropriate adjustment of CP levels is thus essential to balance production efficiency and meat quality in Ningxiang pigs. An increase in the number and size of fat cells can lead to obesity [33]. Furthermore, adipocyte number is determined during early life stages and remains relatively constant throughout adulthood [34,35]. Morphological analysis of abdominal adipose tissue in Ningxiang finishing pigs in this study showed that the LP diet significantly increased the average cross-sectional area, circumference, and diameter of adipocytes and reduced adipocyte density, which was consistent with our expectations. Adipogenesis is the process through which fibroblast-like preadipocytes differentiate into mature adipocytes [33]. The more mature the adipocytes, the larger their area, which suggests that the LP diet increases fat accumulation in adipose tissue by promoting adipocyte maturation, leading to an increase in fat percentage in Ningxiang finishing pigs [36].

Meat quality (mainly including nutritional components, pH value, meat color (L*, a*, and b*), drip loss, and IMF content) is an important economic trait in the pig industry and directly or indirectly affects people’s consumption tendency. The effects of different CP levels on pork quality are still controversial, but the current study showed that different CP levels had no effect on L*, pH_24h_, and drip loss, which is consistent with previous findings [37]. In addition, studies have shown that increasing the level of lysine in the diet of finishing pigs can reduce drip losses [38]. However, the lysine level in our experimental diets was consistent, so there was no difference in drip losses regardless of the dietary CP level. Previous research has shown that meat with higher a* has higher oxidative metabolism [39]. pH value is an important indicator for measuring metabolic changes in meat. Specifically, the increase in glycolysis in meat will lead to accelerated accumulation of lactic acid in muscles, a higher pH drop rate after slaughter, and ultimately a lower pH value, resulting in protein denaturation and reduced meat quality [40,41]. In this study, a* and pH_45min_ in LM of Ningxiang finishing pigs fed the LP diet were higher than those in other treatment groups, indicating that the LP diet may improve meat quality by increasing muscle oxidative metabolism. IMF content is closely related to the tenderness, juiciness, and flavor of meat [42,43]. Research has found that restricting protein levels significantly increased IMF content [37,44]. Our results were in line with these studies, namely that the LP diet significantly increased the IMF content in LM of Ningxiang finishing pigs.

The fatty acid composition in muscle is closely related to the nutritional value of pork. Reducing ΣSFA and increasing ΣMUFA and Σ PUFA in the diet can effectively prevent chronic diseases. Reducing the dietary content of ΣSFA while increasing ΣMUFA and ΣPUFA in pork has been associated with improved nutritional quality of the meat, which may contribute to a reduced risk of chronic diseases in human consumers [45,46]. In addition, the reduction in ΣSFA content and the increase in unsaturated fatty acid content in muscle can improve the flavor of meat [47]. The current study showed that feeding an LP diet significantly increased the ΣMUFA content and reduced the SFA (C23:0) content in LM of Ningxiang finishing pigs. This finding is similar to early reports, which showed that the IMF and ΣMUFA content of meat has increased with the restriction of protein content in the diet [48,49]. C18:1n9c, a MUFA that has been shown to have a beneficial effect on disease recovery, is the main product of de novo lipogenesis, and its concentration increases with increasing IMF content in pigs [50,51]. In this study, oleic acid was the only ΣMUFA in the LM of Ningxiang finishing pigs that was affected by dietary CP level and was the main reason for the difference in ΣMUFA. Moreover, the changing trend of C18:1n9c in LM in this study was completely consistent with the changing trend of IMF content, indicating that the changes in IMF content in LM of Ningxiang finishing pigs with different CP levels may be caused by changes in fatty acid composition in muscle, especially changes in C18:1n9c content. This indicates that limiting dietary CP levels can effectively improve the nutritional value and meat quality of pork.

However, despite enhancing meat quality, dietary CP restriction also led to an increased carcass fat percentage and a reduced lean meat yield. To further elucidate the molecular mechanisms driving these phenotypic changes (particularly the shifts in carcass fat deposition and IMF content) and to inform future nutritional strategies, we subsequently focused our comparative analyses on the HP and LP groups. This choice was guided by two main considerations. First, the HP and LP diets represent the extremes of dietary CP levels, offering a clearer contrast to evaluate the biological impact of CP restriction. Second, the MP group showed phenotypic characteristics very similar to the HP group, limiting its value in revealing dietary CP levels’ effects on the molecular mechanisms of phenotypes. Thus, excluding the MP group allowed for a more focused investigation into the effects of dietary CP restriction on fat deposition and muscle composition. The liver is the major organ for fat and protein metabolism in animals and is very sensitive to changes in dietary CP levels [52]. This study found through liver metabolomics that the LP diet significantly reduced the metabolism of nicotinate and nicotinamide in the livers of Ningxiang finishing pigs. Niacin inhibits hepatic triglyceride synthesis and increases hepatic lipid oxidation [53]. Niacin supplementation at 100 mg/kg has been shown to significantly reduce fat percentage in Ningxiang finishing pigs [1]. Trigonelline, 1-methylnicotinamide, and beta-nicotinamide mononucleotide have the effects of lowering lipids and alleviating obesity [54,55,56,57]. In conclusion, the increase in fat percentage of Ningxiang finishing pigs fed the LP diet may be related to the decrease in the accumulation of metabolites related to niacin and niacinamide metabolism in the liver.

Previous studies have identified several key genes involved in the tryptophan–niacin pathway, including *IDO1*, *IDO2*, *TDO2*, and *NNMT* [54,58]. Acetyl-CoA carboxylase (including two isoforms, ACACA and ACACB) is a key rate-limiting enzyme in lipogenesis [59,60,61]. Furthermore, fatty acid synthase (FASN) is a key protein for de novo fatty acid synthesis, and inhibiting the transcription of *FASN* can inhibit fatty acid biosynthesis [62,63]. We then observed that in the livers of Ningxiang finishing pigs fed the LP diet, the transcript levels of genes involved in the tryptophan–niacin pathway (*IDO2* and *TDO2*) were decreased, while the transcript levels of genes involved in lipogenesis (*FASN*) were increased. In addition, the LP diet had a tendency to increase *ACACA* mRNA expression in the liver of Ningxiang finishing pigs. Previous studies have shown that niacin supplementation can inhibit the expression of FASN and ACAC in bovine mammary epithelial cells, thereby reducing milk fat production [64]. Therefore, it can be speculated that the LP diet increases the fat percentage of Ningxiang finishing pigs by inhibiting tryptophan–niacin metabolism in the liver and promoting the expression of lipogenesis-related genes.

Transcriptome-based WGCNA identified the role of the AMPK signaling pathway in regulating carcass fat percentage and IMF deposition in Ningxiang finishing pigs. AMPK is a key signal regulating lipogenesis. AMPK inhibits the synthesis of cholesterol, triglycerides, and fatty acids through phosphorylation and activates the absorption and β-oxidation of fatty acids [65]. The study by Li et al. [66] showed that reducing dietary CP levels reduced LM weight and inhibited AMPK phosphorylation levels. The study found that reducing dietary CP levels increased IMF deposition in finishing pigs, which may be related to the reduction in p-AMPKα levels [67]. This aligns with the results of the present study; that is, the decrease in p-AMPK in LM of LP diet may improve the content of IMF by promoting adipogenesis and inhibiting fat oxidation.

## 5. Conclusions

In summary, when Ningxiang pigs were fed an MLP (CP: 11.90%) diet during the finishing period, their ADG performed best. Feeding Ningxiang finishing pigs an LP diet increased fat percentage and decreased lean meat percentage, while improving meat quality by elevating a*, pH_45min_, IMF, ΣMUFA, and C18:1n9c, and decreasing C23:0 and C20:3n6. Additionally, the observed increase in fat percentage, IMF, and ΣMUFA in pigs fed an LP diet may be attributed to the inhibition of genes related to tryptophan–niacin metabolism in the liver, upregulation of genes associated with hepatic fat formation, and inhibition of the AMPK signaling pathway in muscle tissue. These findings provide new theoretical insights into optimizing CP levels in Ningxiang pig diets while identifying key metabolic pathways influencing carcass fat accumulation. Future research should focus on developing nutritional strategies to counteract excessive fat deposition in LP-fed pigs, ensuring both optimal growth performance and meat quality.

## Figures and Tables

**Table 1:** Composition and nutrient levels of experimental diets (% of dry matter).

Ingredient	HP	MHP	MP	MLP	LP
Corn, g/kg	590.10	605.80	624.20	642.00	657.50
Soybean meal, g/kg	164.00	127.00	90.00	53.00	15.00
Rice bran meal, g/kg	212.00	230.00	245.00	261.00	280.00
L-Lysine, g/kg	0.00	1.10	2.20	3.30	4.40
DL-Methionine, g/kg	0.00	0.10	0.30	0.40	0.60
L-Threonine, g/kg	0.00	0.50	1.00	1.50	2.10
L-Tryptophan, g/kg	0.00	0.10	0.30	0.40	0.60
L-Valine, g/kg	0.00	0.50	1.10	1.70	2.30
L-Isoleucine, g/kg	0.00	0.50	1.10	1.70	2.30
Calcium carbonate, g/kg	0.30	0.40	0.50	0.40	0.50
Calcium hydrogen phosphate, g/kg	10.60	11.00	11.30	11.60	12.00
Sodium chloride, g/kg	3.00	3.00	3.00	3.00	3.00
Premix ^1^, g/kg	20.00	20.00	20.00	20.00	20.00
Total	1000	1000	1000	1000	1000
**Nutritional level**					
NE ^2^ MJ/kg	9.85	9.85	9.86	9.86	9.86
GE ^3^ MJ/kg	16.92	17.34	17.00	17.22	17.26
CP ^3^ %	15.56	13.99	12.94	11.90	10.31
SID Lys ^2, 4^, %	0.62	0.62	0.62	0.62	0.62
SID Met ^2, 4^, %	0.24	0.24	0.24	0.24	0.24
SID Thr ^2, 4^, %	0.46	0.46	0.46	0.46	0.46
SID Trp ^2, 4^, %	0.12	0.12	0.12	0.12	0.12
SID Val ^2, 4^, %	0.61	0.61	0.61	0.61	0.61
SID Ile ^2, 4^, %	0.46	0.46	0.46	0.46	0.46
Ca ^2^, %	0.49	0.49	0.49	0.49	0.49
STTD P ^2^, %	0.20	0.20	0.20	0.20	0.20

^1^ Supplied per kilogram of diet: Fe, 90 mg; Cu, 3.5 mg; Mn, 3 mg; Zn, 55 mg; I, 0.15 mg; Se, 0.25 mg; vitamin A, 1350 IU; vitamin D3, 160 IU; vitamin E, 15 IU; vitamin K, 0.5 mg; vitamin B_12_, 6 μg, riboflavin, 3.5 mg; D-pantothenic acid, 10.5 mg; biotin, 0.1 mg; folic acid, 0.5 mg; choline, 0.45 g. ^2^ NE, SID Lys, SID Met, SID Thr, SID Trp, SID Val, SID Ile, Ca, and STTD P are all calculated values. ^3^ GE and CP are measured values. ^4^ SID = standardized ileal digestibility. HP = high-protein; MHP = moderate-high-protein; MP = moderate-protein; MLP = moderate-low-protein; LP = low-protein; GE = gross energy; STTD P = standardized total tract digestible phosphorus.

**Table 2:** Primers used for real-time quantitative PCR analysis.

Genes ^1^	Primers	Primers Sequences (5′ to 3′)	NCBI Accession Number
*IDO1*	Forward	GCCTCACCCTTACGATGCTT	NM_001246240.1
	Reverse	TGCTGAGCGTTGCTAACTTC	
*IDO2*	Forward	CCCCGGTGTGGATACTTCAG	XM_021077739.1
	Reverse	TCAAGAGGGGCATCAGAGGA	
*TDO2*	Forward	ACCGGTGGGTCTTCAGGTTA	XM_003128997.6
	Reverse	TCTTCGGTATCCAGTGTCGG	
*NNMT*	Forward	TCATTGCCACCGACTACACG	NM_001123146.1
	Reverse	TGACTCTGTTCCCTTCGAGC	
*FASN*	Forward	CTGCTGGACTCGCTCTTTGA	NM_001099930.1
	Reverse	CTTTGCCTATGTGCTTGCCC	
*ACACA*	Forward	AGCAAGGTCGAGACCGAAAG	XM_021066238.1
	Reverse	TAAGACCACCGGCGGATAGA	
*ACACB*	Forward	CCCGACCATGTTTGTCCTCA	NM_001206399.1
	Reverse	GGTGAGGCGGTAACTGTTGA	
*RPL4*	Forward	CGCTGGTCATGTCTAAAGGTCA	XM_005659862.3
	Reverse	ATTCGGCGACGGTTTCTCAT	

^1^ *IDO1* = indoleamine 2,3-dioxygenase 1; *IDO2* = indoleamine 2,3-dioxygenase 2; *TDO2* = tryptophan 2,3-dioxygenase 2; *NNMT* = nicotinamide N-methyltransferase; *FASN* = fatty acid synthase; *ACACA* = acetyl-CoA carboxylase alpha; *ACACB* = acetyl-CoA carboxylase beta; *RPL4* = ribosomal protein L4.

**Table 3:** Effect of CP levels on growth performance in Ningxiang finishing pigs ^1^.

Items ^1^	HP	MHP	MP	MLP	LP	SEM	*p*-Value		
							ANOVA	Linear	Quadratic
Initial BW, kg	52.19	52.20	53.15	52.10	53.16	0.41	0.914	0.573	0.890
Final BW, kg	77.97	79.26	81.59	80.99	79.66	0.56	0.240	0.193	0.101
ADG, kg/d	0.42 ^c^	0.44 ^abc^	0.46 ^ab^	0.47 ^a^	0.43 ^bc^	0.01	0.038	0.119	0.014
ADFI, kg/d	1.95	1.95	2.00	2.01	1.96	0.02	0.839	0.674	0.470
G:F	0.22	0.22	0.23	0.24	0.22	0.00	0.378	0.348	0.114

^1^ HP = high-protein; MHP = moderate-high-protein; MP = moderate-protein; MLP = moderate-low-protein; LP = low-protein; BW = body weight; ADG = average daily gain; ADFI = average daily feed intake; G:F = ratio of ADG to ADFI; SEM, standard error of the mean; *n* = 5. ^a,b,c^ within a row means that they do not share the same letter and are significantly different by Duncan’s multiple range test (*p* < 0.05).

**Table 4:** Effect of dietary CP levels on carcass characteristics of Ningxiang finishing pigs.

Items	HP	MP	LP	SEM	*p*-Value		
					ANOVA	Linear	Quadratic
Slaughter rate, %	69.85	70.58	70.81	0.46	0.694	0.419	0.806
Leg-to-hip ratio, %	25.65	25.89	24.53	0.01	0.179	0.149	0.233
Carcass straight length, cm	86.64	86.79	86.43	0.67	0.979	0.903	0.870
Carcass oblique length, cm	75.50	76.79	75.93	0.56	0.659	0.766	0.394
Backfat thickness, mm	37.00	37.31	39.58	1.25	0.676	0.423	0.723
Skin rate, %	11.76	10.41	11.36	0.37	0.318	0.658	0.151
Bone rate, %	14.43	14.86	13.26	0.43	0.295	0.266	0.270
Fat percentage, %	36.69 ^b^	35.75 ^b^	40.44 ^a^	0.79	0.027	0.037	0.067
Lean meat percentage, %	37.12 ^ab^	38.98 ^a^	35.37 ^b^	0.55	0.020	0.147	0.013

HP = high-protein; MP = moderate-protein; LP = low-protein; SEM, standard error of the mean; *n* = 7. ^a,b^ within a row means that they do not share the same letter and are significantly different via Duncan’s multiple range test (*p* < 0.05).

**Table 5:** Effect of dietary CP levels on meat quality of Ningxiang finishing pigs.

Items ^1^	HP	MP	LP	SEM	*p*-Value		
					ANOVA	Linear	Quadratic
L* (Lightness)	45.56	45.42	44.51	0.51	0.679	0.423	0.736
a* (Redness)	8.54 ^b^	9.02 ^b^	10.28 ^a^	0.26	0.013	0.004	0.416
b* (Yellowness)	7.06	7.30	7.77	0.17	0.232	0.097	0.734
pH_45min_	6.25 ^b^	6.25 ^b^	6.64 ^a^	0.07	0.026	0.018	0.157
pH_24h_	5.70	5.56	5.62	0.04	0.369	0.411	0.252
Drip loss, %	2.03	1.64	1.92	0.15	0.581	0.779	0.322
IMF, %	7.36 ^b^	6.02 ^b^	13.43 ^a^	1.05	<0.001	0.001	0.003

^1^ HP = high-protein; MP = moderate-protein; LP = low-protein; IMF = intramuscular fat; SEM, standard error of the mean, *n* = 7. ^a,b^ within a row means that they do not share the same letter and are significantly different by Duncan’s multiple range test (*p* < 0.05).

**Table 6:** Effect of dietary CP levels on long-chain fatty acid composition (% of total fatty acids) in the LM of Ningxiang finishing pigs.

Items ^1^	HP	MP	LP	SEM	*p*-Value		
					ANOVA	Linear	Quadratic
C16:0	26.60	26.54	26.95	0.28	0.829	0.631	0.716
C17:0	0.22	0.08	0.20	0.03	0.114	0.736	0.043
C18:0	13.69	13.49	13.10	0.17	0.373	0.176	0.791
C20:0	0.35	0.31	0.32	0.03	0.883	0.757	0.335
C23:0	2.13 ^ab^	2.65 ^a^	1.13 ^b^	0.24	0.020	0.054	0.026
ΣSFA	42.98	43.23	41.70	0.29	0.052	0.052	0.110
C16:1	3.85	3.99	3.89	0.13	0.917	0.898	0.698
C18:1n9t	0.12	0.09	0.21	0.03	0.171	0.229	0.478
C18:1n9c	41.96 ^ab^	40.54 ^b^	44.69 ^a^	0.71	0.038	0.080	0.044
C20:1n9	0.88	1.05	1.04	0.05	0.256	0.167	0.364
ΣMUFA	45.69 ^b^	45.90 ^b^	49.91 ^a^	0.73	0.011	0.007	0.113
C18:2n6c	9.13	9.52	7.39	0.54	0.251	0.201	0.281
C20:3n6	0.40 ^a^	0.48 ^a^	0.24 ^b^	0.04	0.014	0.038	0.022
Σn-6	9.54	10.00	7.64	0.58	0.219	0.184	0.249
C18:3n3	0.17	0.32	0.24	0.06	0.700	0.688	0.394
C20:3n3	0.02	0.15	0.16	0.04	0.301	0.177	0.687
Σn-3	0.20	0.33	0.40	0.05	0.257	0.111	0.241
C20:2	0.40	0.40	0.35	0.02	0.465	0.272	0.589
ΣPUFA	10.14	10.87	8.39	0.60	0.228	0.232	0.208
ΣPUFA/ΣMUFA	0.22	0.24	0.17	0.02	0.188	0.188	0.192
ΣPUFA/ΣSFA	0.24	0.25	0.20	0.01	0.333	0.311	0.278

^1^ HP = high-protein; MP = moderate-protein; LP = low-protein; ΣSFA = total saturated fatty acids; ΣMUFA = total monounsaturated fatty acids; ΣPUFA = total polyunsaturated fatty acids; SEM, standard error of the mean, *n* = 7. ^a,b^ within rows means that they do not share the same letter and are significantly different by Duncan’s multiple range test (*p* < 0.05).

## Data Availability

All RNA-seq data have been submitted to the Sequence Read Archive (SRA) database with accession number PRJNA 1154424.
